# Analysis of Expression Pattern and Genetic Deletion of *Netrin5* in the Developing Mouse

**DOI:** 10.3389/fnmol.2016.00003

**Published:** 2016-01-26

**Authors:** Andrew M. Garrett, Thomas J. Jucius, Liam P. R. Sigaud, Fu-Lei Tang, Wen-Cheng Xiong, Susan L. Ackerman, Robert W. Burgess

**Affiliations:** ^1^The Jackson LaboratoryBar Harbor, ME, USA; ^2^Department of Neuroscience and Regenerative Medicine, Department of Neurology, Medical College of Georgia, Georgia Regents UniversityAugusta, GA, USA; ^3^Howard Hughes Medical InstituteChevy Chase, MD, USA

**Keywords:** dorsal root entry zone, motor exit point, axon guidance, chemorepulsion

## Abstract

Boundary cap cells (BCC) are a transient, neural-crest-derived population found at the motor exit point (MEP) and dorsal root entry zone (DREZ) of the embryonic spinal cord. These cells contribute to the central/peripheral nervous system (CNS/PNS) boundary, and in their absence neurons and glia from the CNS migrate into the PNS. We found *Netrin5* (*Ntn5*), a previously unstudied member of the netrin gene family, to be robustly expressed in BCC. We generated *Ntn5* knockout mice and examined neurodevelopmental and BCC-related phenotypes. No abnormalities in cranial nerve guidance, dorsal root organization, or sensory projections were found. However, *Ntn5* mutant embryos did have ectopic motor neurons (MNs) that migrated out of the ventral horn and into the motor roots. Previous studies have implicated semaphorin6A (*Sema6A*) in BCC signaling to plexinA2 (*PlxnA2*)/neuropilin2 (*Nrp2*) in MNs in restricting MN cell bodies to the ventral horn, particularly in the caudal spinal cord. In *Ntn5* mutants, ectopic MNs are likely to be a different population, as more ectopias were found rostrally. Furthermore, ectopic MNs in *Ntn5* mutants were not immunoreactive for NRP2. The netrin receptor deleted in colorectal cancer (DCC) is a potential receptor for NTN5 in MNs, as similar ectopic neurons were found in *Dcc* mutant mice, but not in mice deficient for other netrin receptors. Thus, *Ntn5* is a novel netrin family member that is expressed in BCC, functioning to prevent MN migration out of the CNS.

## Introduction

Neural crest cells migrate from the dorsal neural tube during vertebrate embryonic development and give rise to a variety of peripheral cell types, including the enteric and autonomic nervous systems, the neurons and glia of the sensory dorsal root ganglia (DRG), and myelinating Schwann cells. Boundary cap cells (BCC) are a transient neural crest-derived cell population that reside adjacent to the embryonic spinal cord at the point where sensory axons enter the spinal cord (the dorsal root entry zone, DREZ) and at the point where motor axons exit the spinal cord (the motor exit point, MEP; Altman and Bayer, 1984; Niederländer and Lumsden, 1996; Golding and Cohen, 1997; Bravo-Ambrosio and Kaprielian, 2011).

The function of BCC has been studied through ablation and lineage tracing experiments, taking advantage of the transcription factor KROX20, which serves as a marker of this cell population (Wilkinson et al., 1989; Golding and Cohen, 1997). In mice, a variety of genetic resources based on *Krox20* (*Egr2*) have been developed, including *Krox20*-driven *Cre* and diphtheria toxin strains. Ablation studies with diphtheria toxin revealed a paucity of TrkA-positive nociceptive neurons in the DRG, suggesting that BCC serve as progenitors that differentiate into these sensory neurons. This is consistent with lineage tracing studies performed with *Cre*-reporters combined with *Krox20-Cre* mice, which also indicated that BCC can become satellite glia in the DRG and proximal Schwann cells in the dorsal root (Maro et al., 2004; Hjerling-Leffler et al., 2005; Aquino et al., 2006). Ventrally, the ablation of BCC causes central glial cells and motor neuron (MN) cell bodies to migrate out of the ventral horn of the spinal cord into the ventral root axons (Vermeren et al., 2003). The deletion of semaphorin6A (*Sema6a*) or its receptors neuropilin2 or plexin-A2 (*Nrp2*, *Plxna2*) partially recreates this MN migration phenotype in mice, and a similar signaling system is involved in chick (Bron et al., 2007; Mauti et al., 2007; Chauvet and Rougon, 2008). Therefore, BCC are not only a transient progenitor population of neural crest cells, they also provide signals contributing to the demarcation of the central vs. peripheral nervous system (PNS).

Netrins are a family of neurodevelopmental signaling molecules that are involved in cell migration and axon outgrowth and guidance, similar to the semaphorins (Ishii et al., 1992; Serafini et al., 1994; Lai Wing Sun et al., 2011). Classical netrins consist of N-terminal laminin-EGF-like domains and a C-terminal netrin domain. They are secreted factors that bind two families of receptors, the UNC5 family (UNC5A-D in vertebrates), and the UNC40 family [deleted in colorectal cancer (DCC) and neogenin in vertebrates; Moore et al., 2007]. Down syndrome cell adhesion molecule (DSCAM) has also been implicated as a netrin receptor (Ly et al., 2008; Liu et al., 2009), although its function as a netrin receptor remains controversial (Palmesino et al., 2012). In addition to the classical netrins, vertebrate genomes also encode two GPI-linked netrins (G-netrins or laminets; Nakashiba et al., 2000, 2002; Yin et al., 2002). Netrin1 is the best studied of the netrin gene family, and is well established for its role in commissural axon guidance. The mouse and human genomes also contain genes designated netrin3, and netrin4, but these genes are less well studied, in part due to more subtle phenotypes following deletion in the mouse genome (Wang et al., 1999; Yin et al., 2000; Hayano et al., 2014). Genome sequencing and homology searches also revealed netrin5, which has not been functionally characterized beyond an analysis of its expression by immunolabeling (Yamagishi et al., 2015).

Here, we report additional characterization of *Netrin5* (*Ntn5*), a netrin gene family member found on mouse Chromosome 7 and human Chromosome 19. This gene encodes a classical netrin that is robustly expressed by BCC during embryonic development. The deletion of *Netrin5* in mice also results in the mis-migration of MNs out of the ventral horn of the spinal cord and into the ventral root. This work extends our understanding of boundary cap cell signaling, and assigns a function to a previously uncharacterized netrin family member.

## Materials and Methods

### Validation of the *Ntn5* Transcription Unit

The predicted *Ntn5* transcript was experimentally verified by reverse transcription and polymerase chain reaction (PCR) using RNA isolated from whole mouse embryos. RNA was prepared by standard Trizol extraction and first strand cDNA was prepared using a combination of random and oligo-dT priming and Super Script III reverse transcriptase (Invitrogen). Many primer combinations were used, but the forward primer GGA GGC CAC TAT GGC GTA GG and reverse GCT GAC AGT ATC TCT GAA GG were particularly informative and spanned the alternative splice site (exon 3). All sequences match those available in genome browsers (mouse GRCm38), with the exception that the longer isoform including exon 3 is not in current gene assembly predictions, as described in the see “Results” Section and Figure S1B.

### Genetic Deletion of *Ntn5*

The *Ntn5* gene was targeted in the mouse genome by standard homologous recombination strategies. A targeting vector was designed to fuse a farnesylated yellow fluorescent protein (YFPF) into the second exon of *Ntn5*. This was done using bacterial recombineering to introduce the YFPF and a downstream neomycin-resistance cassette flanked by FRT recombination sites into the beginning of exon 2, deleting through exon 6 of *Ntn5*, leaving only exons 1 and 7 intact (Lee et al., 2001). The *Ntn5* gene was contained on mouse BAC RP22-513I7, and the following synthetic oligonucleotides were used to generate the YFPF-FRT-Neo-FRT insert that was recombined into the BAC: GGA ATC CTC AGC AGG GTG GAC ACC AAC TGA CCC CAT CTG CC ACCT CTG TCT ACA GGT GCC acc atg tgt agc aag ggc (uppercase-*Ntn5* sequence, underlined-beginning of exon 2, lowercase-YFPF fusion) and GAA GTG GAA GGA TGG GGA AAA GGC AGG CCT GTT TTC CTC TCT CAC TTA CCA TAA TCC TGC Tcg agc cct taa tta acc gg (uppercase-*Ntn5* sequence compliment of exon 6, lowercase-vector downstream of the FRT-Neo cassette). The extent of the *Ntn5* deletion was constrained by the interdigitated *Sec1* gene on the opposite strand. The *Ntn5* targeting vector was electroporated into R1 ES cells and G418 resistant clones were picked and screened for homologous recombination by a PCR assay. Seven of four hundred and fifty clones screened were correctly recombined, and two of these were microinjected into C57BL/6J blastocysts to generate chimeric mice. Germline transmission of the mutation was achieved and homologous recombination was confirmed by Southern blotting of *Hind*III digested genomic DNA from mice. The presence of the neomycin resistance cassette results in the introduction of a new *Hind*III site, reducing the restriction fragment from 6 kb in wild type to 4.5 kb in mutant animals. The neomycin cassette was then removed from the genome by breeding to *Flpe* transgenic mice (Rodríguez et al., 2000). Sequencing confirmed the excision of the neomycin cassette, and that YFPF was in frame with the *Ntn5* start site. Mice were examined for YFPF expression, but this was undetectable by either endogenous fluorescence or by antibody staining, even in BCC at embryonic stages known to express *Ntn5*. Mice were genotyped by PCR. The wild type allele was detected as a 300 bp PCR product from the following primer pair: TTTGGTTTCTGGGTGGCAGT and GAATGTCTGTGCCAGCCTCT. The mutant allele was detected as a 600 bp product from the following pair: GGGCTCAGGCAGGACCAGGA and GTTCACCTTGATGCCGTTCT. An annealing temperature of 60°C was used for both PCR reactions.

### Mouse Strains

All animals were housed in the research animal facility at The Jackson Laboratory under standard housing conditions with a 12:12 light dark cycle and food and water ad libitum. All procedures using animals were performed in accordance with The Guide for the Care and Use of Laboratory Animals and were reviewed and approved by the Animal Care and Use Committee of The Jackson Laboratory. Previously described mouse strains include: *Dscam^−/−^* = B6.CBy-*Dscam^del17/Rwb^* (Fuerst et al., 2008), *Unc5c^−/−^* = B6.cgUnc5c^rcmTg(Ucp)1.23Kz/Slac^ (Ackerman et al., 1997), *Neogenin^−/−^* = B6.129-Neo1, *DCC^−/−^* = B6.129S2-*Dcc^tm1Wbg^*, and *Netrin1^GT^* = B6.129-*Ntn1^Gt(pGT1.8TM)629Wcs^* (Burgess et al., 2006). Roughly equal numbers of mice of either sex were analyzed.

### *NTN5* Expression Constructs

Constructs for expression of netrin5 in mammalian cells were made by cloning the coding sequence of both the short and long isoforms into expression vectors. The AP-tag five vector was used to produce a fusion of NTN5 with alkaline phosphatase and Myc and 6XHis tags at the carboxy terminus (Gene Hunter). A custom vector with expression driven by the RSV promoter was also used to place a Flag-epitope tag at the C-terminus. These constructs expressed robustly in cell lines based on western blotting of cell extracts and immunofluorescence, but recombinant protein was not efficiently secreted into the media for purification. These constructs are available upon request.

### *Ntn5 In Situ* Hybridization

For fluorescent *in situ* hybridization (ISH), digoxygenin labeled riboprobes were made directly from RT-PCR amplification products, in which T3 (5′) and T7 (3′) sequences for *in vitro* transcription were incorporated into the primers to generate sense control and antisense probes respectively. The 5′ primer sequence used was aattaaccctcactaaagggCAGGTGCCGGCTCTACTGTG (lower case is T3, uppercase is *Ntn5*), and the 3′ reverse primer sequence was gtaatacgactcactatagggcCTCACACCCTGG GCCAGTG (T7 lower case, *Ntn5* uppercase), producing an amplicon matching most of exon 2 of the *Ntn5* gene. Fresh frozen sections from E15.5 embryos were cut on a Leica cryostat and hybridized in a formamide buffer according to standard protocols (Schaeren-Wiemers and Gerfin-Moser, 1993; Burgess et al., 2004). Hybridization was detected using the Cy3 tyramide signal amplification system according to manufacturer’s protocols (Perkin Elmer). For the ViewRNA assay, embryos were flash frozen in OCT using 2-methyl-butane in a dry ice/ethanol bath. 12 μm thick cryosections were cut on a Leica cryostat and placed on charged Leica X-tra slides. Slides were shipped to Affymetrix overnight on dry ice. There, the assay was optimized for *Ntn5* and *Krox20* probes and performed according to the manufacturer’s protocol. http://www.panomics.com/products/rna-in-situ-analysis/view-rna-overview. Probes for *Ntn5* were distributed across the sequence encoded by all 7 exons of the *Ntn5* gene except the alternative exon 3.

### Q-RT-PCR

RNA was isolated from whole E15.5 embryos using Trizol reagent and 5 μg of total RNA per sample was used to make cDNA with Superscript III according to the manufacturers’ protocols. Q-RT-PCR was performed in triplicate using a range of sample volumes (0.01–3 μl) with SYBR Green PCR Master Mix on an Applied Biosystems 7500 Real Time PCR System. PCR primers were as follows: *Ntn5* (ACCGACTACAGGACACTCTTT and CTTGCCAACCAACTGGGTC), *Sec1* (GCAGAACTGAGGG AAGACAC and CAGTCTCGGGTGGCATG), *GAPDH* (AGGTCGGTGTGAACGGATTTG and TGTAGACCATGTAGTTGAGGTCA). The relative abundance of each transcript was calculated by the DDCt method in comparison to *GAPDH*. Primers spanned introns to reduce possible genomic DNA contamination, with *Ntn5* primers matching sequence in exons 1 and 2, and *Sec1* primers matching sequence in exons 1 and 3.

### General Phenotyping

Mice were analyzed by a number of phenotyping tests according to the Knockout Mouse Phenotyping (KOMP2) Project pipeline described here: www.jax.org/research-and-faculty/tools/knockout-mouse-project/phenotyping. Tests include SHIRPA, grip strength, open field, light/dark, holeboard exploration, acoustic startle/prepulse inhibition, electrocardiogram, glucose tolerance, body composition, eye dysmorphology survey, auditory brainstem response, electroretinography, seizure threshold, and sleep patterns. Nine adult homozygous mutants were analyzed (6 females and 3 males) along with 10 control animals (5 females and 5 males).

### Sensory Phenotyping

#### Von Frey Testing

Subjects were acclimated to individual arenas atop a wire mesh grid for a 60 min habituation period. A standard set of calibrated microfilaments (0.02–2.0 g) was applied to the plantar surface of the hindpaw, starting with the left side and beginning with the microfilament in the middle of the series (0.4 g). The microfilament was applied until it bowed and was maintained in place for 3 s or until the paw was withdrawn. A positive response was scored as a withdrawal from the microfilament. Upon a positive response, the next lower microfilament was tested, consistent with the “up-down” method (Chaplan et al., 1994). If no response was observed, then the next higher microfilament in the series was applied. The right paw was tested immediately after the completion of assessment of the left paw. Paw withdrawal threshold (g) is defined as the minimum force applied resulting in withdrawal of the paw.

#### Thermal Nociception

Subjects were acclimated to the testing room for a 60 min habituation period under standard lighting conditions. Mice were then placed into a clear enclosure directly atop a hot plate analgesia meter (IITC Life Sciences) constructed of a black anodized, aluminum plate (275 mm × 263 mm × 15 mm) heated to a constant temperature of 52 ± 0.2°C for a maximum test time of 30 s. A trained observer, blind to genotype, recorded via stopwatch timer, the latency of hindpaw withdrawal as indicated by observation of a hindpaw flick, lick, or jump. For both sensory tests, 17 mutants were analyzed (9 females and 8 males) with eight controls (4 females and 4 males).

### Immunocytochemistry

#### Cryosections

E13.5 embryos were isolated, eviscerated, and immersed in 4% PFA for 1 h on ice. The embryos were then rinsed three times for at least 1 h in PBS and cryopreserved by sinking in 30% sucrose. Neonatal spinal columns were isolated, fixed overnight by immersion in 4% PFA at 4°C, and cryopreserved in 30% sucrose. Tissue was then frozen in OCT on dry ice and sectioned at 12 μm on a Leica cryostat. Slides were blocked for 1 h at room temperature with 2.5% BSA in PBS with 0.5% Triton-X-100 in a humidified chamber, then incubated with primary antibody diluted in blocking solution overnight at 4°C. After rinsing in PBS, slides were incubated with fluorescently conjugated secondary antibodies and lectins diluted in PBS for 1 h at room temperature. Slides were thoroughly rinsed in PBS with DAPI added to the final rinse. Then, coverslips were mounted using Dako fluorescence mounting media. Images were collected on a Zeiss AxioImager.Z2 equipped with Zeiss AxioCam MRm monochrome CCD camera.

#### Whole Mount Embryos

Whole mount neurofilament staining was performed as in Burgess et al. (2006). Briefly, E11.5 embryos were isolated and fixed overnight in 4% PFA, then washed in 0.9% NaCl. Embryos were dehydrated in a methanol series up to 80%, and then incubated for 3 h in 3% H_2_O_2_/Dent’s fixative (80% Methanol 20% DMSO) followed by 3 h in TBST (1% Tween). Embryos were incubated for 2 days at room temperature in primary antibody (2H3, 1:500) diluted in TBST with 5% milk powder and 5% DMSO, followed by extensive washing in TBST. Secondary antibody (HRP conjugated anti-mouse) diluted in TBST/milk/DMSO was applied overnight at room temperature followed by more extensive washing in TBST. HRP-conjugated secondary antibody was detected using a DAB substrate kit from Molecular Probes according to the manufacturer’s protocol. Samples were dehydrated through a methanol series and cleared with methyl salicylate. Embryos were imaged with a Leica Wild M10 microscope equipped with a Leica DFC 300 FX cooled-CCD color camera.

#### Cell Culture

HEK293 cells were cultured at 37°C in DMEM (Hyclone) supplemented with 10% FBS (Gibco) and 100 U/ml of penicillin G and streptomycin (Gibco). For transient transfection, cells were transfected with indicated plasmids using polyethylenimine (Sigma-Aldrich). In brief, cells were cultured in 6-well plates to 50% confluence, and then incubated with precipitates formed by 3 μg of plasmid DNA and 1 μl of polyethylenimine (5 mg/mL). After 8~12 h of incubation with PEI/DNA polyplex, media was replaced with the fresh serum-containing medium. At 48 h after initial transfection, cells were fixed with 4% PFA (paraformaldehyde) with 4% sucrose at room temperature for 15 min, permeabilized with 0.3% TritonTX-100, and blocked with 10% goat serum and 0.1% saponin. Primary antibodies used were rabbit anti-Flag (Sigma, 1:1,000), and mouse anti-myc (Developmental Studies Hybridoma Bank (DSHB), 1:1000). The colocalization was quantified using ImageJ software.

#### Antibodies

Primary antibodies used were as follows: mouse anti-Islet 1/2 (DSHB, clone 39.4D5, 1:500), mouse anti-HB9 (DSHB, clone 81.5C10, 1:500), mouse anti-neurofilament-M (DSHB, clone 2H3, 1:500), rabbit anti-neurofilament (Sigma, 1:500), rabbit anti-Krox20 (Covance, 1:200), rabbit anti-CGRP (Millipore, 1:250), rabbit anti-parvalbumin (Chemicon, 1:500), rabbit anti-GFAP (Sigma, 1:500), rabbit anti-neuropilin-2 (Cell Signaling, 1:250), chicken anti-βGal (Aves, 1:1000). Secondary antibodies were Alexa-fluor conjugates (AF488, AF594, and AF647, Life Technologies, 1:500). Isolectin-B_4_ was used at 1:200 (Life Technologies).

#### Image Analysis

For sensory neuron quantification, cryosections roughly through the center of DRGs were stained and imaged and the number of CGRP, IB_4_, and neurofilament stained cells were counted. Means per DRG were compared by student’s *t*-test. For quantification of ectopic MNs, every tenth section was collected along the entire length of the spinal column. In every section where a ventral root was clearly visible exiting the spinal cord, the number of Islet 1/2-positive cells in the root was counted. The mean number of ectopic cells per root was compared across animals by student’s *t*-test (*Ntn5* mutants vs. controls) or by ANOVA with Tukey’s post-hoc test (receptor mutants).

## Results

### The *Ntn5* Gene Encodes a Classic Netrin

**Figure 1:**
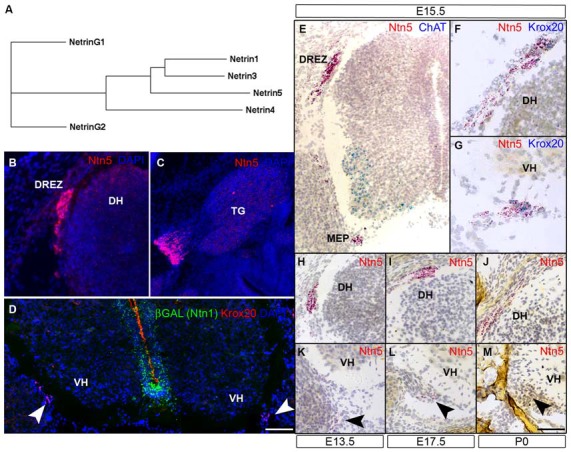
***Ntn5* is expressed in Boundary cap cells (BCC).** A dendrogram calculated from the primary amino acid sequences of mouse netrins demonstrates that netrin5 is more similar to netrin1 and netrin3 than is netrin4 **(A)**. *Ntn5* expression was assayed by *in situ* hybridization. Using a riboprobe and tyramide signal amplification, expression was detected at the dorsal root entry zone (DREZ) of the spinal cord **(B)** and adjacent to the trigeminal ganglia **(C)** at embryonic day 15.5. By the ViewRNA branched DNA probe technique, expression was verified at the DREZ and detected at the motor exit point (MEP, motor neurons (MNs) labeled for ChAT, **E**). *Ntn5*-expressing cells at both locations were *Krox20*-positive BCC **(F,G)**. Expression at the MEP was strongest between E13.5 and E17.5 **(K,L)** and was barely detectable at P0 **(M)**. Likewise, labeling at the DREZ peaked between E13.5–E17.5 **(H,I)** and was diminished by P0 **(J)**. In *Ntn1^GT/+^* embryos immunostained for βGal—a reporter of *Ntn1* expression in these mice—there is no colocalization with Krox20-positive BCC **(D)**. TG, trigeminal ganglion; DH, dorsal horn of the spinal cord; VH, ventral horn. Scale bar is 550 μm in **(B,C,E)**, 300 μm in **(F,G)**, and 400 μm in **(D,H–M)**.

Netrin5 (*Ntn5)* was first found in a search of mouse genomic sequence and expressed sequence tags (ESTs) using a BLAST search for netrin-related molecules. Mouse *Ntn5* is comprised of seven exons on mouse Chromosome 7 (7:45,684,022–45,694,556, GRCm38), and a homolog with an identical genomic structure is found in a region of shared synteny in the human genome on chromosome 19 (19:49,164,664–49,176,338, GRCh37). We empirically verified the transcripts produced by the *Ntn5* gene using RT-PCR of RNA from E15.5 embryos and sequencing (GB accession number KU356771). Two splice variants were found at roughly equal abundance, and differed by the inclusion or exclusion of the third exon (Figures S1A,B). These mRNAs produce proteins of 452 amino acids (corresponding to CCDS39957) and with the inclusion of exon 3, 515 amino acids. At the amino acid level, the long isoform of netrin5 is 32% percent identical and 46% similar to mouse netrin1, suggesting it is a member of the classical netrin family. The netrin5 primary sequence is more closely related to netrin1 and netrin3 than is netrin4 (Figure 1A). The gene is expressed at low levels; we could detect both transcripts by RT-PCR from embryos and muscle, but not by northern blot (Figure S1C).

### *Ntn5* is Expressed in Boundary Cap Cells

*Ntn5* expression was recently described in the neurogenic regions of the adult brain (Yamagishi et al., 2015), but its expression pattern during development is unknown. To investigate this, we performed *in situ* hybridization (ISH) by two methods. First, we performed fluorescence ISH by standard techniques using digoxygenin-labeled riboprobes and tyramide signal amplification. In transverse sections of E15.5 mouse embryos, we found distinct labeling adjacent to the DREZs of the spinal cord, the point at which sensory axons project into the CNS. In sagittal sections, we detected expression just proximal to the trigeminal ganglia of the fifth cranial nerve (Figures 1B,C). No signal was detected using sense control probes (not shown). To verify and expand on this expression pattern, we used an independent ISH approach (Affymetrix Quantigene ViewRNA assay). This method uses branched DNA oligonucleotides and direct signal amplification, and has the sensitivity to detect modestly expressed transcripts with very low background. Consistent with our fluorescent ISH findings, we observed strong labeling at the DREZ. We also detected expression adjacent to the MEP of the spinal cord, where motor axons project to the periphery (Figure 1E). Expression was detectable at E11.5 (not shown) and was maintained through P0, but was strongest between E13.5 and E15.5 (Figures 1H–M). The *Ntn5*-expressing cells were reminiscent of BCC. Indeed, in multiplexed ISH sections, the *Ntn5* expressing cells also labeled positively for *Krox20*, a BCC marker (Figures 1F,G). We also examined embryonic sagittal sections and found very limited *Ntn5* expression in other areas, but signals were detected in structures directly adjacent to the CNS, suggestive of a role in cranial nerve guidance (Figure S2).

Netrin5 is part of a gene family, which led us to look for *Ntn1* expression in BCC to determine if there may be functional redundancy with this well characterized netrin. *Ntn1* expression has not been reported in BCC, and we did not find *LacZ* expression in BCC in heterozygous *Ntn1* mice carrying a gene trap reporter (Figure 1D), suggesting that *Ntn5* expression in these cells is not redundant with *Ntn1*.

### Generation of *Ntn5* Mutant Mice

**Figure 2:**
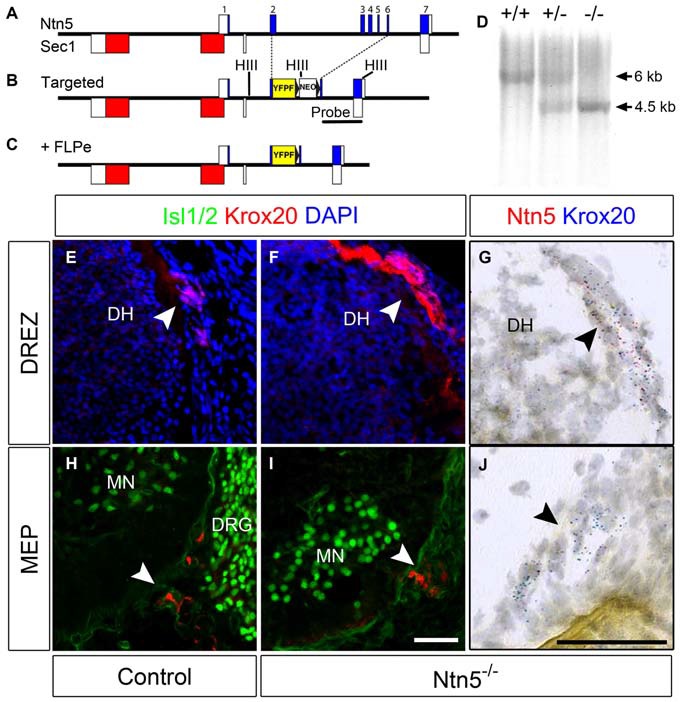
**BCC develop in the absence of *Ntn5*.** *Ntn5* was deleted without disrupting the *Sec1* gene on the opposite strand. A cassette encoding a farnesylated YFP and a FRT-flanked neomycin resistance gene was knocked into the *Ntn5* locus so that YFP was fused to the 5th base pair of exon 2, while the remainder of exons 2–6 was deleted. Open boxes indicate untranslated regions of exons **(A,B)**. Subsequent breeding to FLPe-expressing mice removed the neomycin cassette **(C)**. Mice were genotyped using primers to exons 3 and 4 to detect wild type alleles and with primers to intron 1 and GFP to detect the mutant allele, **(A,C)**. Homologous recombination was verified by Southern blot analysis using a probe (indicated in **B**) to detect a Hind*III* fragment (sites in **B**, HIII) shifted from 6–4.5 kb **(D)**. Krox20-positive BCC were still present at the DREZ and MEP in *Ntn5* mutant embryos, as detected by immunoflouorescence and *in situ* hybridization (E13.5, **E–J**), while Ntn5 transcripts detected by ISH were virtually eliminated **(G,J)**. DH, dorsal horn of the spinal cord; MN, motor neurons; DRG, dorsal root ganglia. Scale bar is 50 μm.

The exons of *Ntn5* interdigitate on the opposite strand with those of *Sec1*, a gene encoding a fucosyltransferase enzyme (Figure 2A). In order to knockout *Ntn5* without disrupting *Sec1*, we targeted exons 2–6, which reside in the first intron of *Sec1* (Figures 2B,C). By homologous recombination, we replaced these exons with a cassette encoding a farnesylated YFP fused to the 5′ sequence of exon 2, and a neomycin resistance gene for ES cell selection flanked by FRT sites (see “Materials and Methods” Section). Given that the deleted exons encode more than 70% of the *Ntn5* open reading frame, we presume the mutation effectively creates a null allele. Consistent with this, in homozygous mutant mice, there was no reduction in *Sec1* expression as measured by Q-RT-PCR, while *Ntn5* expression was undetectable (Figure S3). Expression of *Ntn5* was also effectively eliminated in BCC based on *in situ* hybridization (Figures 2G–J), but Krox20-positive BCC persisted in their normal location in the absence of *Ntn5* with no obvious reduction in cell number or clustering (Figures 2E–J). Unfortunately, the farnesylated YFP reporter was not visible either natively or with immunostaining, even after FLPe-mediated excision of the neomycin cassette. We verified by sequencing that the YFP was in frame to form a fusion after the first 13 amino acids of NTN5.

Homozygous mutant mice (*Ntn5^−/−^*) were overtly normal and indistinguishable from wild type littermates in size, life span, and fertility. We analyzed a cohort of *Ntn5^−/−^* mutants using a battery of phenotyping tests developed for the KOMP2 project and did not find any significant deviations from control values (see “Materials and Methods” Section).

### Netrin5 Prevents Motor Neuron Cell Body Migration into Ventral Roots

**Figure 3:**
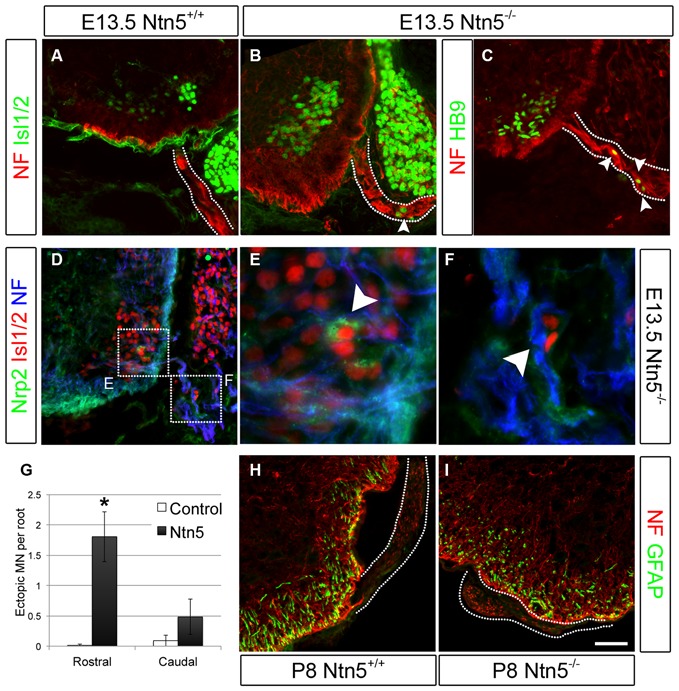
**Netrin5 keeps MNs cell bodies from migrating out of the CNS.** Immunolabeling of E13.5 embryos showed ectopic MN cell bodies in the ventral roots of *Ntn5^−/−^* mutants. Ectopias were not observed in controls **(A)**, but cells positive for Islet 1/2 **(B)** and HB9 **(C)** were observed in mutants, indicative of MNs. While some MNs were positive for nrp2, **(D,E)** ectopic MNs in mutant embyros were not **(D,F)**, suggesting that these MNs are a different population from those known to use a semaphorin6A/nrp2 mechanism. Furthermore, there were more ectopias in the rostral half of the embryo than in the caudal (quantified in **G**) which is the inverse of what was observed in *Nrp2* mutant embryos. CNS glia such as GFAP-positive astrocytes did not enter the ventral root **(H,I)** indicating this aspect of the CNS/PNS boundary was not lost. NF, neurofilament. Scale bar is 50 μm. *indicates *P* < 0.05 by student’s *t*-test.

*Krox20*-positive BCC were still present in *Ntn5^−/−^* mutants (Figures 2D–F). Therefore we reasoned that *Ntn5^−/−^* mutants would display only a subset of the phenotypes found in mice in which BCC were ablated. Previous studies in mice with boundary cap cell ablation found that MN cell bodies migrated along their axons out from their normal position in the ventral horn of the spinal cord into the ventral roots (Vermeren et al., 2003). A similar phenotype was observed in mice lacking *Sema6a*, or its receptors plexin-A2 and neuropilin-2, or in chicks with knockdown of related semaphorin pathway genes (Bron et al., 2007; Mauti et al., 2007). These previous studies defined ectopic MNs in the ventral root as cell bodies that labeled positively for MN markers including Isl1/2 and HB9, and assumed that these cell migrated from the ventral horn based on the known contribution of BCC to the CNS/PNS boundary and as the most expedient explanation for their source. We are therefore using the same analyses and interpretation here. Interestingly, in *Sema6a* or *Nrp2* mutant embryos, there were far more ectopic MNs in the caudal aspect of the embryo at the level of the hindlimbs than rostrally at the level of the forelimbs (Bron et al., 2007). To assess MN cell body placement, we stained transverse cryosections of E13.5 embryos for MN markers (Islet 1/2 and HB9), along with neurofilament to identify the ventral root axons. Indeed, we observed many instances of ectopic MN cell bodies that had migrated out of the ventral horn and into the root (Figures 3A–C). To ask if these ectopic MNs were the same subset of cells that depend on *Sema6a* signaling to remain in the ventral horn, we systematically quantified the number of ectopic MNs in the rostral and caudal halves of the embryo. In contrast to the previously reported semaphorin results, we saw far more ectopias in the rostral aspect (mean 1.8 ectopic MNs per root, *SD* = 0.7) than at the caudal end (0.5/root, *SD* = 0.4, Figure 3G). In rostral roots, 63% of the roots showed ectopic MN cell bodies with an average of 2.5 cells per root (thus an average of 1.8 per root). These numbers are comparable to the frequency observed in caudal roots in the *Sema6a* or *Nrp2* knockout embryos (Bron et al., 2007). Furthermore, while some MNs were strongly labeled for neuropilin-2, these remained restricted to the ventral horn in the *Ntn5^−/−^* embryos, and the ectopic MNs were not nrp2 positive (Figures 3D–F). Ectopic MNs were observed in each of 12 mutant embryos and quantification is based on 5 mutant animals.

In terms of the impact of this phenotype on motor performance, our general phenotyping of these mutants included tests of grip strength and general motor activity (see “Materials and Methods” Section). By all measures *Ntn5*^−/−^ mutants were within the expected parameters for C57BL/6 mice. We have extensive expertise characterizing mice with overt motor phenotypes (Seburn et al., 2006; Achilli et al., 2009; Wooley et al., 2009; Burgess et al., 2010; Motley et al., 2011), and found no qualitative gait defect in *Ntn5*^−/−^ mice.

In addition to MN cell bodies exiting at the ventral root, glia from the CNS can aberrantly migrate into the periphery in the absence of BCC (Kucenas et al., 2009; Coulpier et al., 2010). We did not observe this in *Ntn5^−/−^* mutant embryos: GFAP-positive astrocytes remained restricted to the spinal cord (Figures 3H,I).

### DCC is a Possible NTN5 Receptor

**Figure 4:**
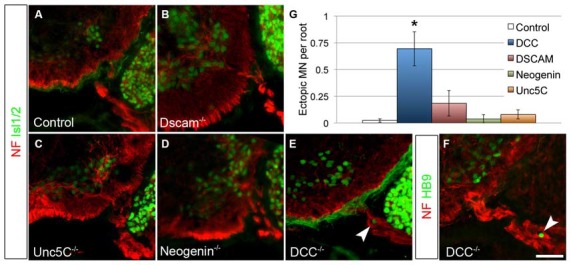
**DCC is required to prevent ectopic motor neuron migration.** A series of netrin receptor mutant embryos were analyzed at E13.5 for ectopic MNs in the rostral half of the spinal cord. As in controls (**A**, *n* = 3), ectopias were not observed in *Dscam* (**B**, *n* = 3), *Unc5C* (**C**, *n* = 3), or *Neogenin* (**D**, *n* = 2) mutants. There were, however, ectopic MNs in *DCC* mutants (*n* = 4) positive both for Islet 1/2 **(E)** and HB9 **(F)**. Quantified in **(G)**. Scale bar in **(F)** is 50 μm. *indicates *P* < 0.05 by Tukey’s *post hoc* test.

As a classical netrin, NTN5 likely acts through the known repertoire of netrin receptors. To ask which receptor is responsible for restricting MN cell bodies to the ventral horn, we analyzed E13.5 mutant embryos lacking *Dcc*, *Neogenin*, *Dscam*, or *Unc5C*, all four of which are expressed by MNs (Burgess et al., 2006; Liu et al., 2009). No ectopias were observed in *Unc5C* or *Neogenin* mutant embryos, and very rarely in *Dscam* mutant embryos (Figures 4A–D). We did, however, find ectopic MN cell bodies positive for both Islet 1/2 and HB9 in the rostral ventral roots of *Dcc* mutant embryos (*mean* = 0.7/root, *SD* = 0.27, Figures 4E,F). These were not as frequently observed as in *Ntn5^−/−^* mice, but were significantly more numerous than in controls or any of the other receptor mutants (Figure 4G). Like in *Ntn5^−/−^* embryos, ectopic neurons were not found in every root, but were observed in 42% of roots, with an average 1.7 cells per root. Thus, the reduced average number of ectopic cells is a product of both fewer ectopic cells per root and fewer roots with ectopic cells.

To assess the potential interaction between netrin5 and DCC, we co-transfected HEK293 cells with Myc- or Flag-tagged DCC or neogenin along with Flag-tagged netrin5 or myc-tagged netrin1. We reasoned that increased colocalization would indicate interaction. Netrin5 showed more colocalization with DCC (Pearson’s correlation = 0.3607, *SEM* = 0.04611) than with neogenin (PC = 0.1894, *SEM* = 0.02917). The colocalization between netrin5 and either receptor was much less than that observed between the netrin1 and DCC (PC = 0.7037, *SEM* = 0.03133) or neogenin (PC = 0.6299, *SEM* = 0.03868). When transfected into astrocytes or HEK293 cells, netrin5 was detected at the cell surface by live cell staining (data not shown), but it was not efficiently secreted into the media when transfected in three separate cell lines, thus precluding the opportunity to directly test binding to DCC.

### Dorsal Roots Develop Normally Without Netrin5

**Figure 5:**
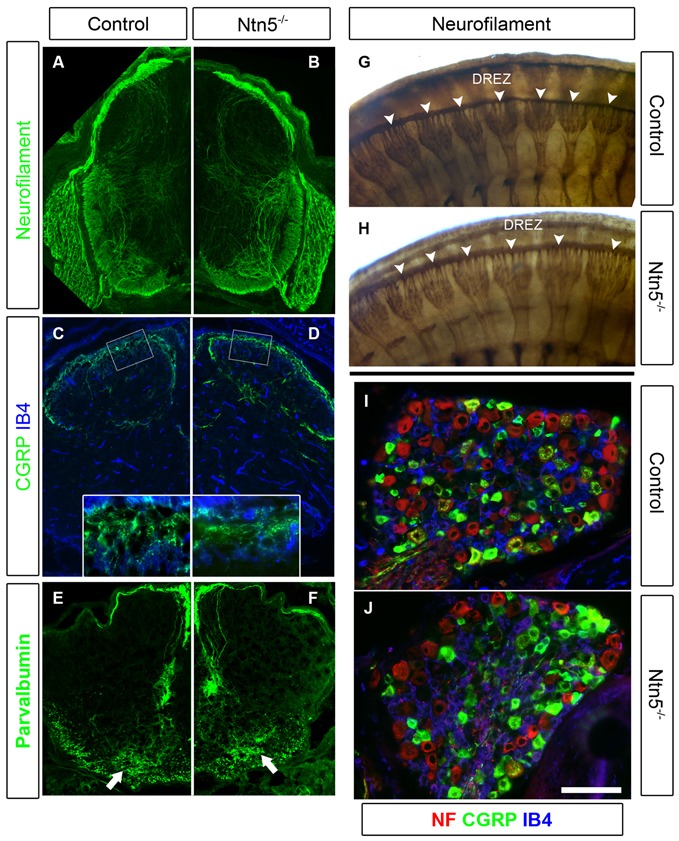
**Sensory neurons project through the DREZ without *Ntn5*.** There was no obvious disorganization of dorsal roots in *Ntn5^−/−^* mutants visualized by immunolabeling for neurofilament in cryosections from E13.5 embryos **(A,B)**, or in whole embryo labeling at E11.5 (**G,H**, arrowheads). Nociceptive fibers positive for CGRP and IB_4_ projected into the superficial laminae at P3 in mutants and controls **(C,D)**, and parvalbumin-positive Ia afferents targeted to the motor neuron region normally (**E,F**, arrows). There were also normal numbers of CGRP- and IB_4_-positive sensory neurons in mutant DRGs **(I,J)**. Scale bar is 400 μm in **(A−F)**, 100 microns in insets **(C,D)**, and 100 μm in **(I,J)**.

Since *Ntn5* is also expressed in dorsal BCC, and ablation of dorsal BCC causes defects in both DRG cell populations and projections into the DREZ, we analyzed DRGs and their sensory inputs into the dorsal spinal cord in the *Ntn5*^−/−^ mice. Dorsal BCC differentiate into sensory neurons, including calcitonin gene related peptide (CGRP) and isolectin B_4_ (IB_4_) positive nociceptive fibers, satellite glia, and the Schwann cells that myelinate the dorsal root (Maro et al., 2004; Hjerling-Leffler et al., 2005; Aquino et al., 2006; Zujovic et al., 2011). In BC-cell-ablated mice there are significantly fewer sensory neurons in the DRG, and in animals mutant for *Sema6a—*a gene expressed in BCC—dorsal roots are severely disorganized (Maro et al., 2004; Mauti et al., 2007). Therefore, we analyzed the general organization of dorsal roots by staining for neurofilament (Figures 5A,B,G,H), as well as the projections of CGRP-positive and IB_4_-positive nociceptive fibers and parvalbumin-positive Ia afferent fibers into the spinal cord in cryosections (Figures 5C–F). We did not find abnormalities in the organization or terminal projections of these fibers. We counted sensory neurons from cryosections through the center of DRGs in mutants and controls (Figures 5I,J). *Ntn5^−/−^* mice had an average of 61.0 IB_4_-positive neurons per DRG (*SD* = 10.9) vs. 62.9 in controls (*SD* = 16.2). CGRP-positive cells were similarly unaffected in mutants (54.8 ± 11.1 *SD* in mutants vs. 62.6 ± 15.8 *SD* in controls), as were strongly neurofilament-positive cells (71.1 in both genotypes, *SD* = 27.0 and 28.8 for mutants and controls). *N* = 8–13 DRGs total from four mice per genotype.

While there were no gross defects in sensory projections into the spinal cord or in the numbers of nociceptive sensory neurons, it remained possible that there were subtle defects that could be functionally relevant. Therefore, we tested adult mice for functional nociceptive defects using von Frey testing and thermal nociception analysis. In von Frey testing, a measure of mechanical sensitivity, *Ntn5^−/−^* mutants exhibited a minimum paw withdrawal threshold at 0.85 g (*SD* = 0.34) compared to 0.89 g in controls (*SD* = 0.33). We used a hot plate test of thermal nociception in which we measured the latency of hind paw withdrawal from a hot plate held at 52°C. The latency in mutants was 14.6 s (*SD* = 4.2) and in controls was 14.2 s (*SD* = 3.2). *N* = 17 mutants and eight littermate controls; the values were within the range expected for C57BL/6 mice. Thus, there were no functional nociceptive defects severe enough to register with either of these tests.

### Netrin5 is not Required for Cranial Nerve Guidance

**Figure 6:**
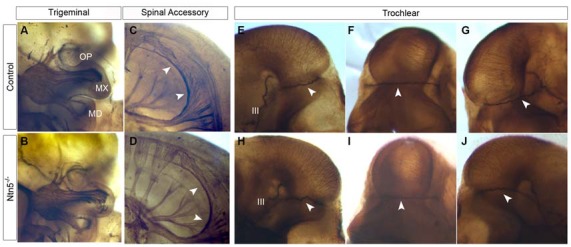
**Cranial nerves develop normally in *Ntn5^−/−^* mutants.** To visualize cranial nerves, whole embryos were stained for neurofilament at E11.5. The three branches of the trigeminal nerve were positioned normally in both controls **(A)** and mutants **(B)**. The three branches are labeled as OP (ophthalmic) MX (maxillary) and MD (mandibular). Likewise, the spinal accessory nerve was not visibly reduced and exhibited normal pathfinding in *Ntn5^−/−^* mutants **(C,D)**. Cranial nerve IV, the trochlear nerve, extended dorsally to cross at the back of the head to innervate the contralateral side in controls **(E−G)** and mutants **(H−J)**. The oculomotor nerve (III) is also visible in these images. All observations were verified in five mutant and five control embryos.

Based on its expression adjacent to the trigeminal ganglia (Figure 1C) and the known functions of netrin guidance systems in the trochlear nerve, spinal accessory nerve, and ophthalmic branch of the trigeminal nerve, we hypothesized that netrin5 may also play a role in cranial nerve guidance (Colamarino and Tessier-Lavigne, 1995; Burgess et al., 2006; Dillon et al., 2007). To assess this, we analyzed E11.5 embryos stained in whole mount for neurofilament-M. The trigeminal nerve lacks its ophthalmic branch in *Neogenin* mutant mice (Burgess et al., 2006). We analyzed this nerve in *Ntn5^−/−^* mutants, and found that the ophthalmic, mandibular, and maxillary branches exhibited normal morphology and targeting (Figures 6A,B). The spinal accessory nerve is severely diminished without netrin1 or DCC or UNC5C (Dillon et al., 2005, 2007), but developed normally in the absence of netrin5 (Figures 6C,D). In *Unc5c* mutants, the trochlear nerve fails to project dorsally and cross to the contralateral superior oblique muscle, but rather innervates on the ipsilateral side. This misprojection is not found in *Ntn1* mutants (Serafini et al., 1996; Burgess et al., 2006), and we did not observe it here in *Ntn5^−/−^* mice (Figures 6E–J).

## Discussion

Here, we provide the first functional characterization of a vertebrate *Netrin5* gene. *Netrin5* is a conserved member of the netrin gene family that is more similar to the classical netrins netrin1 and netrin3 than is netrin4, thus firmly placing it in the classical netrin clade based on amino acid sequence. The genomic organization and primary sequence are well conserved in the mouse and human genomes. *Ntn5* is expressed in BCC during embryonic development, and genetic deletion studies indicate that it functions in ventral BCC to prevent the aberrant migration of MN cell bodies out of the ventral horn of the spinal cord and into the ventral root. *Ntn5* does not have a clear role in dorsal BCC or in sensory neuron development. DCC is a candidate receptor for NTN5 based on a partial recapitulation of the MN migration phenotype in *Dcc* knockout embryos. *Ntn5* has other points of expression and may have other functions in mice, but homozygous knockout animals perform normally in a battery of behavioral tests and are overtly normal, suggesting that any additional functions are likely either to impact very specific cell populations or to be quite subtle. Nonetheless, our findings expand the functional netrin gene family and have several interesting implications for neurodevelopmental signaling pathways.

The boundary between the CNS and PNS is not well understood, but a demarcation between the CNS and PNS clearly exists. Cranial and spinal MNs as well as preganglionic autonomic neurons are the only cell bodies in the CNS that project axons into the periphery. Similarly, cranial nerve and DRG sensory neurons are the only neuronal cell bodies in the periphery that project axons into the CNS. Distinctions such as myelination by oligodendrocytes in the CNS vs. Schwann cells in the PNS further support the presence of such a boundary, in this case possibly established through the interaction of the two different glial cell types (Kucenas et al., 2009). The structural boundary at the MEP and DREZ is not as pronounced as are other boundaries in the nervous system, such as that between the retina and the optic nerve (Elkington et al., 1990), but the physical boundary is functionally important here as well. Radial glia endfeet form a glial limitans around the spinal cord with gaps at the MEP and DREZ where the axons traverse (Fraher et al., 2007). Disruption of these glial endfeet results in cells from the CNS entering the PNS (Lee and Song, 2013).

**Figure 7:**
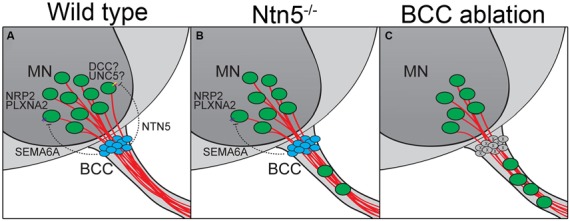
**BCC prevent motor neuron exit through multiple mechanisms. (A)** BCC express SEMA6A, which signals through PLXNA2/NRP2 receptors on motor neurons (MN; Bron et al., 2007; Mauti et al., 2007). NTN5 from BCC prevent ectopic MN migration through putative interactions with DCC, probably in concert with an UNC5 receptor. In the absence of NTN5 **(B)** a subset of MNs exit the CNS. This is a separate subset from that depending on semaphorin signaling for proper positioning. When BCC are completely ablated **(C)**, more MNs enter the ventral root than in *Ntn5* or Sema/Plxn/Nrp mutants alone (Vermeren et al., 2003).

In addition to this structural border, molecular signaling and chemorepulsion also contribute to the boundary. The position of BCC makes them excellent candidates to produce such signal. Indeed, the ablation of BCC results in a migration of MNs out of the ventral horn of the spinal cord, and this has been ascribed to actions of *Sema6a* in BCC and semaphorin receptors plexinA2 and neuropilin2 in the MNs, based on genetic studies in mice and similar findings in chick (Vermeren et al., 2003; Bron et al., 2007; Mauti et al., 2007). Our results indicate that netrin5, potentially signaling in part through DCC, has an identical function in a different population of MNs (Figures 7A,B). That these populations are distinct is suggested by more MNs being mis-positioned in rostral ventral roots in the absence of *Netrin5*, whereas more MNs were mis-positioned in caudal ventral roots in *Sema6a* and *Nrp2* knockout embryos (Bron et al., 2007). MNs in the ventral roots of *Ntn5* mutant embryos also did not label with an anti-neuropilin2 antibody above background levels, whereas many MNs that remained in the ventral horn were positive, again suggesting the netrin5-sensitive MNs are not the same as those responding to SEMA6A. It is worth noting that even when BCC are completely ablated, only a subset of MNs exit the ventral horn (Figure 7C; Vermeren et al., 2003). This, combined with the knockout phenotypes, suggests that signaling systems beyond *Ntn5* and *Sema6A* and that cell types beyond BCC function to restrict MNs to the spinal cord.

Genetic analysis implicates DCC as potentially involved in NTN5 signaling, since *Dcc* mutant mice have a similar MN migration phenotype. No such phenotype was observed for *Neogenin*, *Unc5c*, or *Dscam*, although redundancy or compensation may have masked a role in NTN5 signaling for these proteins. DCC/netrin interactions are typically chemoattractant, although they can be chemorepellant in cooperation with UNC5 family member receptors (reviewed in Moore et al., 2007; Lai Wing Sun et al., 2011). *Unc5C* is not required to prevent MN ectopias based on the phenotype of *Unc5c* mutant embryos. The expression pattern of *Unc5d*, studied using a lacZ-gene trap reporter in heterozygous animals, indicates that it is not expressed in the spinal cord, suggesting it is not involved (data not shown, Kim and Ackerman, unpublished). Unfortunately, *Unc5b* and –*d* mutant embryos die prior to motor axon outgrowth, and therefore the loss of function phenotype cannot be examined. Like *Dcc*, *Unc5A* is expressed in embryonic MNs and is therefore a strong candidate for interacting with DCC to transduce a repellant netrin5 signal (Burgess et al., 2006; Williams et al., 2006).

The signaling functions of netrins and BCC in dorsal sensory neuron projections remain less clear. Following ablation of BCC, there is a paucity of nociceptive neurons in the DRG (Maro et al., 2004). This may represent a failure of signaling for migration or differentiation, or the loss of a progenitor pool resulting in depletion of the daughter population. Consistent with the latter interpretation and with lineage tracing studies, BCC can give rise to DRG neurons, satellite glial cells and myelinating Schwann cells *in vivo* and *in vitro* (Maro et al., 2004; Hjerling-Leffler et al., 2005; Aquino et al., 2006). However, a signaling function for dorsal BCC is suggested by disorganization of dorsal roots in chick embryos following knockdown of *Sema6A* (Mauti et al., 2007). *Ntn5* does not appear to serve a comparable role dorsally, as we were unable to identify changes in the DRG, in the projection of axons into the dorsal horn of the spinal cord, or in sensory function in mice lacking *Ntn5*. Semaphorins and netrins may also be more redundant in their functions in organizing DREZs in mice.

Netrin1 also has a role at the dorsal CNS/PNS boundary. In mice mutant for *Ntn1* or *DCC*, axons from spinal interneurons extend out through the DREZ and into the DRG (Laumonnerie et al., 2014). Furthermore, dorsal-laterally expressed netrin1 signaling through UNC5C affects sensory axon ingrowth into the dorsal spinal cord, specifically contributing to the “waiting period,” during which sensory axons enter the superficial layers of the dorsal horn and project rostro-caudally for roughly 48 h before making layer-specific projections into the dorsal horn itself (Watanabe et al., 2006). SEMA5B serves a similar function in chick (Liu et al., 2014). Based on our results, netrin5 does not appear to be involved in this process, although a subtle difference in timing or a specific subpopulation of sensory axons could easily have been missed in our analyses. There may also be redundancy with other netrin family members, although netrin1 is not expressed by BCC based on our study (Figure 1D) and previous reports (Kennedy et al., 1994, 2006; Williams et al., 2006).

The studies presented here indicate that *Ntn5* is a member of the netrin gene family that is expressed by BCC, and the loss-of-function phenotype is consistent with previous studies on boundary cap cell-mediated activities. Like other netrins, *Ntn5* has a role similar to semaphorin signaling pathways, but is not redundant with those pathways, because it operates in a different population of cells. Netrin5 also appears to signal at least in part through the known netrin receptor DCC. Therefore, this work expands our knowledge of BCC, netrins, and the complementarity of neurodevelopmental signaling systems.

## Funding

The Scientific Services at Jackson are supported in part by NCI Cancer Center Support (CA034196), and KOMP2 is supported by HG006332. Additional funding was provided by the Edward Mallinckrodt, Jr. Foundation and NIH grants NS054154 and NS085285 (RWB), NIH training and postdoctoral fellowship grants T32NS051112 and F32EY021942 (AMG), and an Institutional Development Award (IDeA) from the NIH, GM103423 (in support of LPRS). SLA is an investigator in the Howard Hughes Medical Institute and was supported by NIH grant NS035900. W-CX is supported by NIH AR045781.

## Conflict of Interest Statement

The authors declare that the research was conducted in the absence of any commercial or financial relationships that could be construed as a potential conflict of interest.
