# Supplementary material for: Analysis of Expression Pattern and Genetic Deletion of Netrin5 in the Developing Mouse
Source: Front Mol Neurosci. 2016 Jan 26;9:3. doi: 10.3389/fnmol.2016.00003 (PMC4726805; doi:10.3389/fnmol.2016.00003)
Supplement: Supplementary file 1 [file Image_1.pdf]

Supplementary Material

Analysis of Expression Pattern and Genetic Deletion of *Netrin5* in the Developing Mouse.

Andrew M. Garrett, Thomas J. Jucius, Liam P.R. Sigaud, Fu-Lei Tang, Wen-Cheng Xiong, Susan L. Ackerman, Robert W. Burgess*

*** Correspondence:** Robert.Burgess@jax.org

Supplementary Figures

A

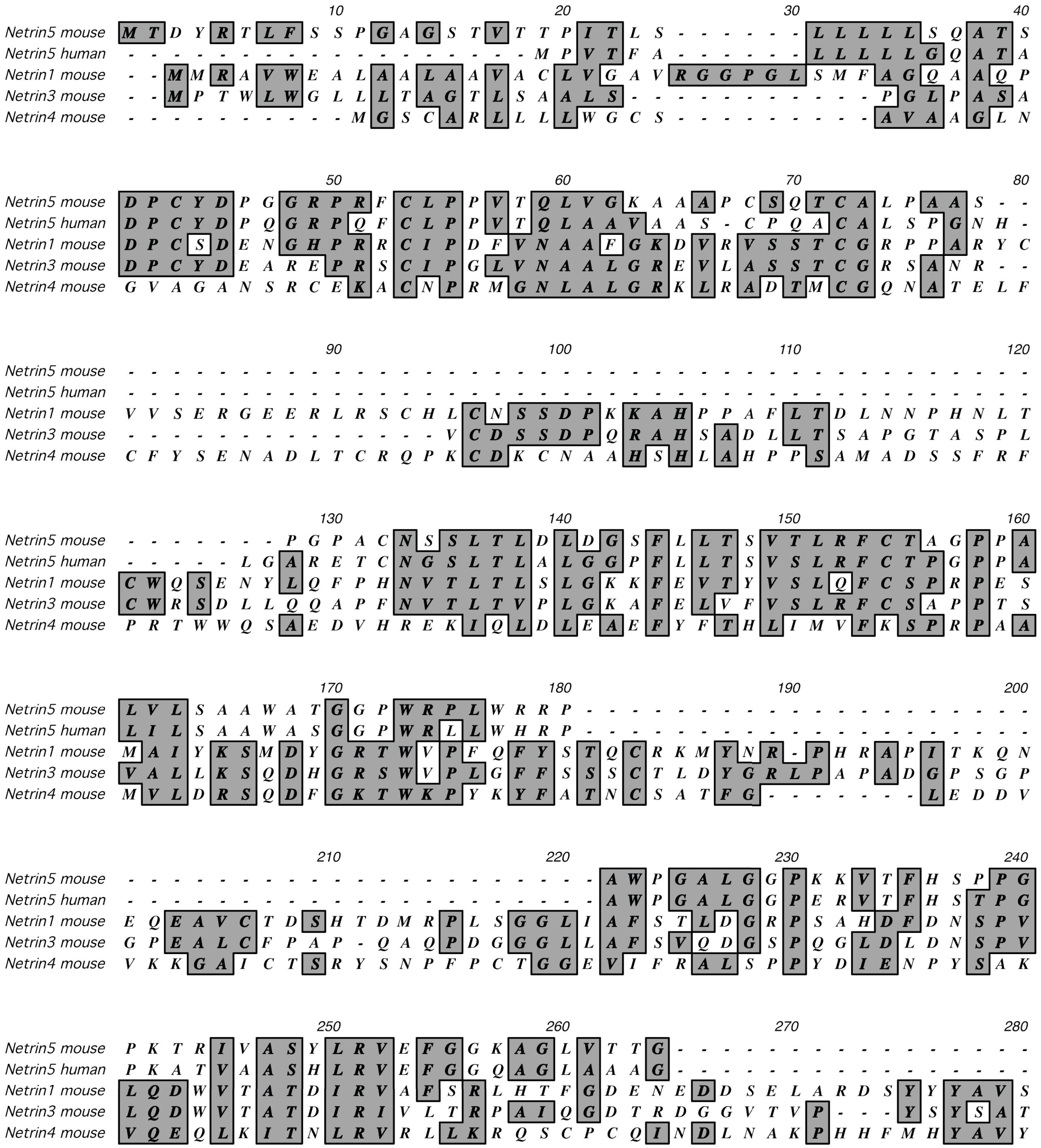

```
                290                 300                 310                 320
Netrin5 mouse   ---VRGRCQCHGHAARCATRAQ-------------PPRCR
Netrin5 human   ---LRGRCQCHGHAARCAARAR-------------PPRCH
Netrin1 mouse   DLQVGGRCKCNGHAARCVRDRDD------------SLVCD
Netrin3 mouse   ELQVGGRCKCNGHASRCLLDTHG------------HLVCD
Netrin4 mouse   DFIVKGSCFCNGHADQCLPVEGFRPIKAPGAFHVVHGRCM

                330                 340                 350                 360
Netrin5 mouse   CRHHTTGPGCESCRPSHRDWPWRPATPQH--PHPCLPCSC
Netrin5 human   CRHHTTGPGCESCRPSHRDWPWRPATPRH--PHPCLPCSC
Netrin1 mouse   CRHNTAGPECDRCKPFHYDRPWQRATARE--ANECVACNC
Netrin3 mouse   CQHGTEGPDCSRCKPFYCDRPWQRATGQE--AHACLACSC
Netrin4 mouse   CKHNTAGSHCQHCAPLYNDRPWEAADGRTGAPNECRTCKC

                370                 380                 390                 400
Netrin5 mouse   NQHARRCRFNSELFRLSGGRSGGVCERCRHHTAGRHCHYC
Netrin5 human   NQHARRCRFNSELFRLSGGRSGGVCERCRHHTAGRHCHYC
Netrin1 mouse   NLHARRCRFNMELYKLSGRKSGGVCLNCRHNTAGRHCHYC
Netrin3 mouse   NGHARRCRFNMELYRLSGRRSGGVCLNCRHNTAGRHCHYC
Netrin4 mouse   NGHADTCHFDVNVWEASGNRSGGVCNNCQHNTEGQHCQRC

                410                 420                 430                 440
Netrin5 mouse   QPGFWRDPSQPITSHKACRACQCHPIG------ATGGMCN
Netrin5 human   QPGFWRDPSQPIFSRRACRACQCHPIG------ATGGTCN
Netrin1 mouse   KEGFYRDMGKPITHRKACKACDCHPVG------AAGKTCN
Netrin3 mouse   REGFYRDPGRVLSDRRACRACDCHPVG------AAGKTCN
Netrin4 mouse   KPGFYRDLRRPFSAPDACKACSCHPVGSAILPFSSVTFCD

                450                 460                 470                 480
Netrin5 mouse   QTSGQCSCKLGVTGLTCNRCGPGYQ---------------
Netrin5 human   QTSGQCTCKLGVTGLTCNRCGPGYQ---------------
Netrin1 mouse   QTTGQCPCKDGVTGITCNRCAKGYQ---------------
Netrin3 mouse   QTTGQCPCKDGVTGLTCNRCAPGFQ---------------
Netrin4 mouse   PSNGDCPCKPGVAGPHCDRCMVGYWGFGDYGCRPCDCAGS

                490                 500                 510                 520
Netrin5 mouse   ---------QSRSPRMPCQRIPEATTTPATT-PVASRSDP
Netrin5 human   ---------QSRSPRMPCQRIPEATTTLATT-PGAYSSDP
Netrin1 mouse   ---------QSRSPIAPCIKIPVAPPTTAAS-SVEE--PE
Netrin3 mouse   ---------QSRSPVAPCVKTPVPGPTEESS-PVE---PQ
Netrin4 mouse   CDPLTGDCISSNADVDWYHEVPAFHSMHNKSEPSWEWEDE

                530                 540                 550                 560
Netrin5 mouse   QCQGYCNVS-----VSSVHMSLQRYCQQDYVLHAQVSASS
Netrin5 human   QCQNYCNMS-----DTRVHMSLRRYCQQDHVLRAQVLAS-
Netrin1 mouse   DCDSYCKAS-----KGKLKMNMKKYCRKDYAVQIHI-LKA
Netrin3 mouse   DCESHCRPA-----RGSYRISLKKFCRKDYAVQVAVGARG
Netrin4 mouse   QGFSALRHSGKCECKEQVLGNPKAFCGMKYSYVLKIKILS
```

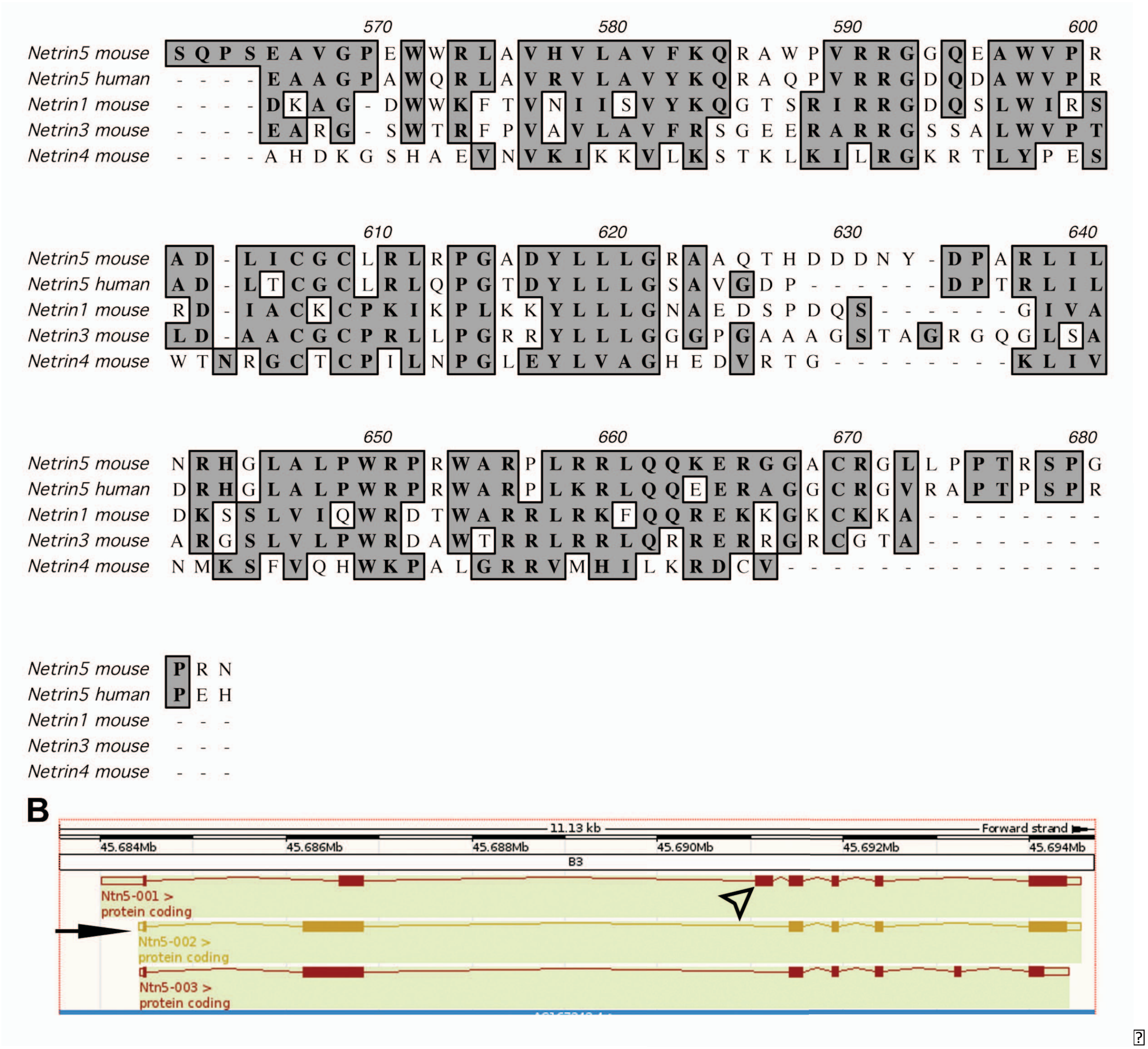

Figure S1: Netrin5 is a classic Netrin. Protein sequence alignment shows a high level of similarity between Netrin5 and other classic Netrins. Similar or identical residues are boxed. Sequence corresponding to the exon omitted in the short transcript is highlighted in blue (**A**). The gene structure of mouse *Ntn5* from the Ensembl genome browser (**B**). The *Ntn5*-002 gene structure was experimentally validated. A second alternatively spliced isoform that also includes exon 3 of the *Ntn5*-001 gene model introduced into the *Ntn5*-002 transcript was also found. Other transcripts corresponding to gene prediction models may exist at low levels, but they were not found experimentally or in EST databases. NCBI gene and transcript models XM_006540887.2, XP_006540950.1 and XM_006540890.2, XP_006540953.1 do correctly predict the long and short Ntn5 transcripts we identified, respectively.

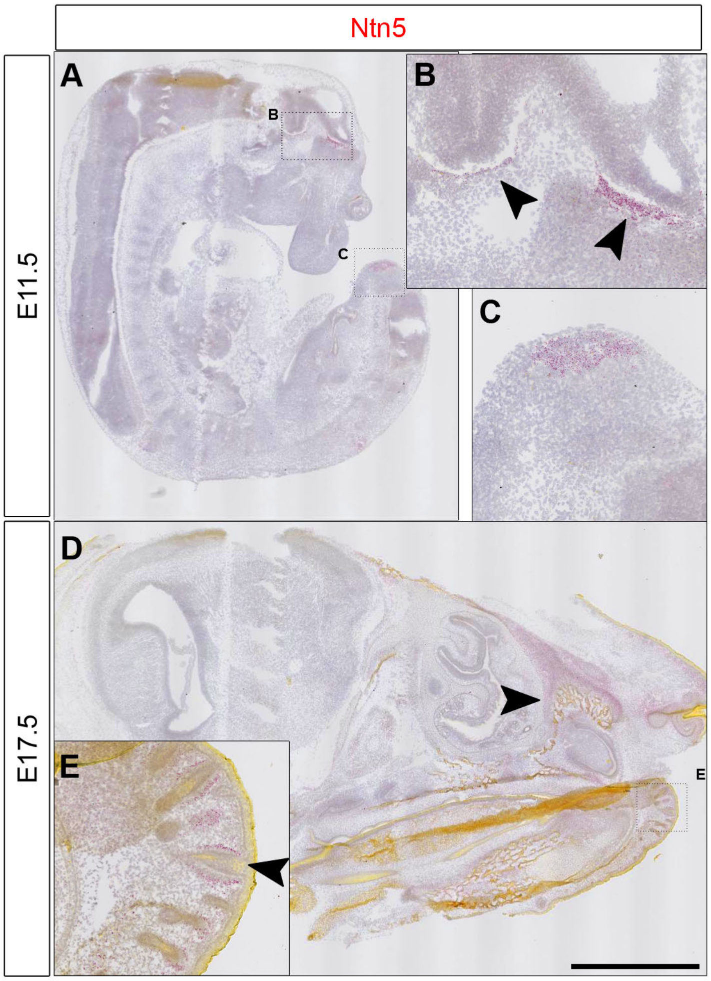

Figure S2: Other *Ntn5* expression. Netrin5 is expressed adjacent to the brain at E11.5 (**A**,**B**) and E17.5 (**D**). There was also expression associated with the olfactory system (**B**,**D** arrow), at the tail (**A**,**C**) and at the base of whiskers in the chin (**D**,**E**). Scale bar is 1.5 mm in **A**,**D** and 400 mm in **B**,**C**,**E**.

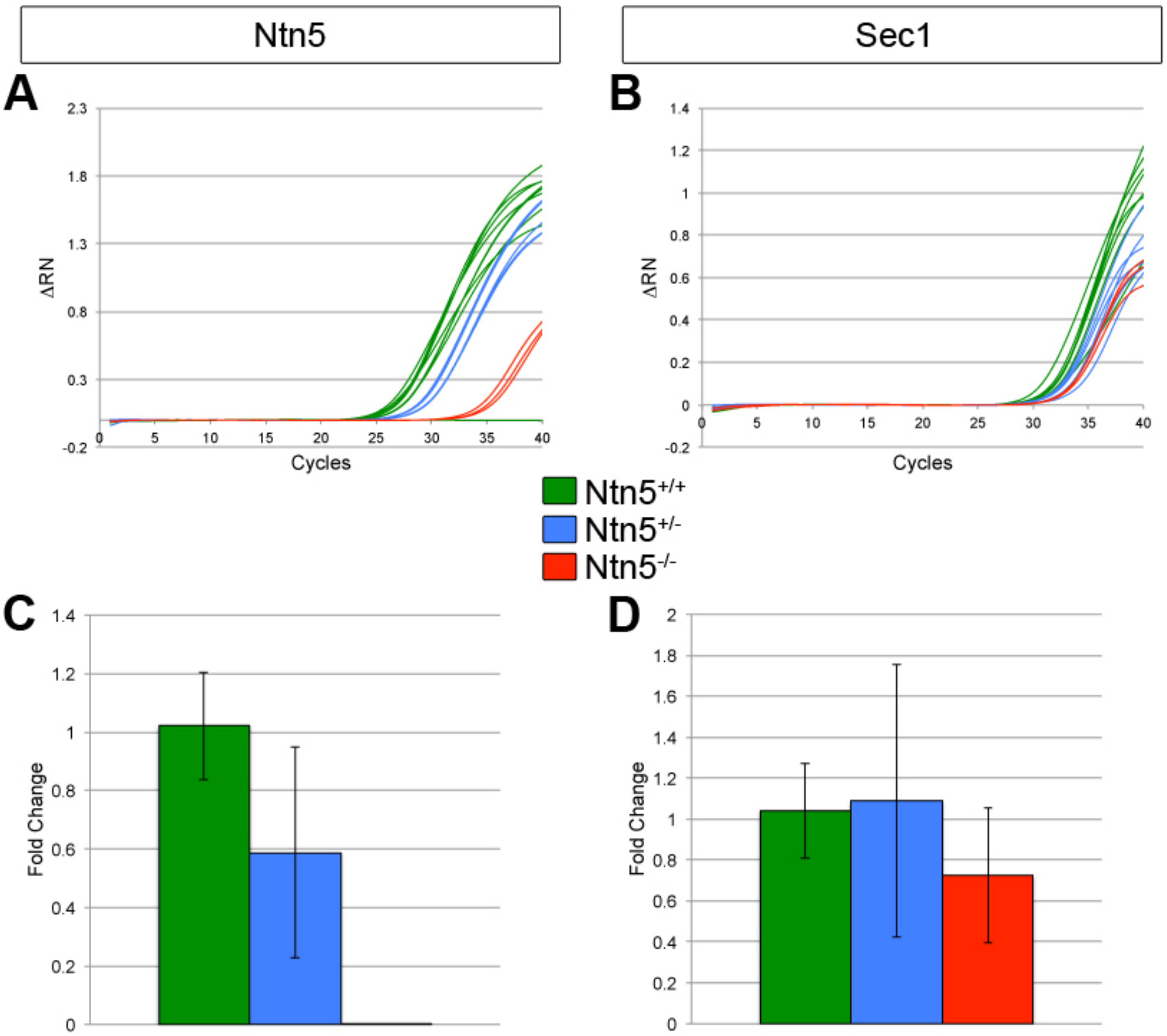

Figure S3: *Ntn5* expression abolished without disrupting *Sec1* expression in Ntn5$^{-/-}$ embryos. QPCR analysis of Ntn5 and Sec1 transcripts from E13.5 *Ntn5*$^{+/+}$, *Ntn5*$^{+/-}$, and *Ntn5*$^{-/-}$ whole embryos demonstrates that Ntn5 transcripts were effectively removed in null mutants (**A**,**C**), while Sec1 transcripts were not significantly reduced (**B**,**D**). Dissociation curves of the Ntn5 products demonstrated that late-arising PCR products from *Ntn5*$^{-/-}$ samples were different from the normal product in *Ntn5*$^{+/+}$ and *Ntn5*$^{+/-}$ samples. **A** and **B** show individual reactions from multiple embryos in triplicate, while fold change values in **C** and **D** are means ± s.e.m. by genotype.
